# Randomised Control Trial Comparing Cypermethrin-Based Preparations in the Prevention of Infectious Bovine Keratoconjunctivitis in Cattle

**DOI:** 10.3390/ani10020184

**Published:** 2020-01-22

**Authors:** Jennifer Allan, Steven Van Winden

**Affiliations:** 1 The Royal Veterinary College, Regional Veterinary Centre South of England, Stinsford Business Centre, Kingston Maurward College, Dorchester DT2 8PY, UK; 2 Royal Veterinary College, Hawkshead Lane, Hatfield, Hertfordshire AL9 7TA, UK; svwinden@rvc.ac.uk

**Keywords:** cypermethrin, infectious bovine keratoconjunctivitis, pour-on preparations, Impregnated ear tags

## Abstract

**Simple Summary:**

Infectious bovine keratoconjunctivitis (IBK) caused by the bacteria *Moraxella bovis* is commonly seen in the summer months spread by face flies causing a painful eye disease. This trial investigated the difference in number of cases of IBK between two groups of animals that were treated with two different fly control products, one a pour-on and one an impregnated ear tag. The growth rate per day was also investigated between animals with cases and those without and between the treatment groups. The trial enrolled 197 animals. Cases of IBK and growth rate were recorded over the grazing season (April–November 2018). Fifty-four cases of IBK were recorded. There was no difference in number of cases between the two treatment groups and there was no difference in growth rate between animals that had a case and animals that had not. Animals that had white faces, which in this trial were Hereford cattle and also the animals under 12 months old were found to be more likely to get a case. Overall, there was no significant difference between the two fly control preparations in preventing IBK, younger animals and white-faced breeds are significantly more likely to suffer from IBK.

**Abstract:**

Infectious bovine keratoconjunctivitis (IBK) caused by *Moraxella bovis* is commonly seen in the summer months spread by face flies. This trial investigated the difference in incidence of IBK cases from natural exposure between two groups of animals, one treated with Cypermethrin pour-on preparation (PON, n = 98) and one with Cypermethrin impregnated ear tags (TAG, n = 99). Daily Live Weight Gain (DLWG) difference was investigated between animals with cases and those without and between treatment groups. A randomised positive control study, enrolled 197 animals split into two treatment groups. Cases of IBK and DLWG were recorded over the grazing season (April–November 2018). Fifty-four cases of IBK were recorded. There was no association between the two treatment groups (*p* = 0.362) and case status. Breed and under 12 months old were significant factors for having a case; (OR 2.3, *p* = 0.014 and OR 3.5, *p* < 0.001 respectively). There was no difference in DLWG between animals that had a case and animals that had not (*p* = 0.739) or between the two treatment groups (*p* = 0.215). Based on our results, there is no significant difference between PON or TAG preparations in the prevention of IBK. Younger animals and white-faced breeds are significantly more likely to suffer with IBK.

## 1. Introduction

Infectious bovine keratoconjunctivitis (IBK) is one of the most common ocular infectious diseases to affect cattle in the UK. The bacterium involved, *Moraxella bovis* (*M. bovis*), is a Gram-negative opportunistic pathogen and can be found on the conjunctiva and in nasal and ocular secretions. In the lead to the clinical presentation of IBK, multiple factors are involved including environmental factors; U.V. light, dust, fly population, other pathogens present and host factors; age, breed and immunity [1]. Young animals and breeds with unpigmented conjunctiva, for example Herefords, are at an increased risk of infection. Unpigmented conjunctiva is more easily damaged by U.V. light and animals in their first grazing season are likely to not have encountered *M. bovis* previously [2].

Transmission can be from ocular secretions in infected cattle to other cattle through fomites or mechanical vectors, most commonly face flies [3,4,5]. Outbreaks occur more commonly in the summer months due to an increase in fly population and also U.V. light exposure. U.V. light causes degeneration of epithelial cells in the cornea and female flies disrupt the conjunctiva when feeding; both allow the *M. bovis* to establish an infection [1]. Once infection is established, the increase in ocular secretions attracts more flies, creating a vicious cycle. *M. bovis* can survive on the legs of flies for up to three days [6], allowing them to transmit bacteria to many animals in a group quickly. 

Mortality from this disease is low, but the morbidity is high with the most common complaint being reduced growth rates over the grazing season [7], resulting from painful ocular lesions. More recently, studies have looked at end weights at weaning and found up to a seven kilo difference [8]. Cattle have a spectrum of clinical signs, from epiphora, lacrimation, blepharospasm, corneal oedema to ulceration; if not treated, ulcers can rupture, leading to prolapse of the anterior chamber and formation of ‘Popeye’ calves [9,10,11]. Associated costs of the disease come from poor growth rates from the associated pain and loss of vision, the direct veterinary costs for treatment and if eye is terminally affected from ulceration, poor aesthetics could also affect market prices.

Fly control has long been the focus of IBK prevention. There are many products licenced for fly control and in different formulations. Topical treatment is the most widely used; however, there are some variations within these preparations. Pour-on preparations are applied to the animal’s skin along its back repeated every four to six weeks and fly tags are applied to the ear the same way as an identification ear tag and slowly release the substance over time. Both have been trialled to varying successes but have not been directly compared against each other [12,13,14]; in particular, there is no recent trial work into the effectiveness of these tags in the UK.

The objectives of this study were to investigate the difference in cumulative incidence of IBK cases between two groups of animals, one treated with a Cypermethrin alpha pour-on preparation and one with Cypermethrin impregnated ear tags. Daily live weight gain (DLWG) difference was investigated between animals that had cases and those without and compared between treatment groups. An economic analysis was also done for the two treatments.

## 2. Materials and Methods

### 2.1. Study Design

A randomised prospective positive control study was used to compare the number of cases of IBK and growth rates of calves split into two groups; Ear tag (TAG) and Pour-on (PON) group. Randomisation was done by assigning TAG or PON as animals came through the crush; alternating animals were assigned the same treatment.

The TAG calves were given two Cypermethrin impregnated ear tags (Flectron Tag, containing 935 mg Cypermethrin [15]) on turnout. The PON calves were treated with Alphacypermethrin pour-on preparation at 10 mL per animal (Dysect Cattle 15 g/L Pour-on Solution, containing alphacypermethrin [16]) every six weeks. Ear tags were removed from TAG calves at sale or housing.

Animals were enrolled to the study on turnout between 27 April 2018 and 8 May 2018, and followed for the whole grazing season. Animals had to be a minimum of 100 kg and have no existing eye lesions. They were allocated to six grazing groups containing between 23 and 51 animals. The groups were stratified by age but not breed. Within each group, PON and TAG were allocated randomly as described above. Animals were weighed at turnout and at either sale or housing. Three groups were sold in September and the others were brought in for housing in November. Overall, 197 animals were enrolled between the ages of three months to 20 months. Breeds included British Friesian (BF), Hereford crosses (HEX) and Swedish Red (SR). All were treated with a long-acting macrocyclic lactone anthelmintic preparation on turnout to control for parasitic gastroenteritis (Cydectin 10% LA containing 100 mg/mL Moxidectin [17]). The animals remaining after September had a repeated dose of anthelmintic (Animec, containing 10 mg/mL Ivermectin). All animals were rotated regularly across paddocks; three of the younger groups were fed concentrates for part of the grazing season, once a day whilst they were checked. No other supplementary feed was given to the other groups. Calves were not vaccinated against IBK. Costs for products used were also analysed for each treatment.

The study was approved by the RVC’s ethical review committee approval board (URN 2018 1805-3). All products used in the trial were licensed for use in cattle in the UK. The trial was conducted on one farm in North Dorset, England, UK. To confirm the presence of IBK on the farm, two affected animals were swabbed and cultured before treatment. Both results were positive for Moraxella sp. and due to the clinical signs and farm history it was not thought necessary to swab each new case. To detect a 20% difference in case incidence significant at the 5% level with 80% power, the trial required 198 animals (split between treatment groups).

### 2.2. Case and Data Management

All groups were kept within a mile radius from the farm; all paddocks had similar levels of fly challenge, being surrounded by woodland and hedgerows. Animals were checked daily by the stockman and any clinical signs of IBK were recorded in a paper diary to be later transferred to an Excel spreadsheet (Microsoft). Descriptive statistics and statistical tests were undertaken in SPSS (IBM SPSS Statistics 25). Multivariate linear models were used to look at risk factors for IBK cases (logistic regression) and DLWG (linear regression). We also evaluated cases in animals under 12 months old as a subset, using the same outcomes as the complete dataset, risk factors for IBK cases and DLWG differences.

The researcher checked animals weekly alongside the stockman. A ‘case’ was defined as an animal being observed to have any of the clinical signs of IBK: epiphora, excess lacrimation, blepharospasm, corneal oedema or ulceration. IBK cases were treated with amoxicillin topical eye ointment (Orbenin). If the case did not respond within seven days, there was a repeat treatment of a sub conjunctival injection into the dorsal eyelid conjunctiva with an Oxytetracycline (Alamycin) and dexamethasone (Colvasone) combination (1 mL of each in one syringe) was given. The farmer was made aware of minimum withhold time periods as this was an off-licence treatment.

## 3. Results

Overall, 197 animals were enrolled, three died over the study period and two were missed in the final weighing session. There were 115 animals under 12 months old at turnout. There were overall 111 BF, two SR and 84 HEX, details of each group are below (Table 1). As SR only had two animals, this breed category was excluded.

Despite not managing the 198 animals needed by the sample size calculation. The level of difference able to be detected with 197 animals was 20.5%.

### 3.1. Case Incidence

There were 54 cases of IBK recorded over the trial period. The number of cases of IBK in the TAG group were 24 (44%, n = 54) and cases in the PON group were 30 (56%, n = 54). Two calves had both eyes affected and five animals had repeat cases; repeat cases were included only once in the analysis. There was no significant difference in number of cases (24 vs. 30 respectively) between the two treatment groups TAG and PON (OR 0.7, 95% CI 0.4–1.4, *p* = 0.362, n = 195). 

Multivariate regression showed that Hereford animals and being under 12 months old were significant factors for having a case; (OR 2.3, 95% CI 1.2–4.4, *p* = 0.014 and OR 3.5, 95% CI 1.7–7.3, *p* < 0.001 respectively). The gender and management group had no influence on case incidence (OR 1.0, 95% CI 0.5–1.9, *p* = 0.927 and OR 0.9, 95% CI 0.6–1.2, *p* = 0.459, respectively) (Table 2).

### 3.2. Daily Live Weight Gain 

In total, 190 animals were included in this analysis (Table 3) due to three dying during the study period (one of which had a case) and two were missed in the final weighing session. In total, 95 animals from the older groups were weighed at turnout and then sold in September 2018. In total, 95 animals in the younger groups were kept over the winter and weighed at turnout, then housing in November 2018. The two month difference between the end weight of these animals and thus time contributing to DLWG calculation was not a significant factor on DLWG (*p* = 0.236).

### 3.3. Animals under 12 Months Old 

Due to the high number of cases in animals under 12 months (78%, n = 54), these were evaluated in closer detail (n = 113). There were 42 cases with 29 being in HEX (n = 50) and 13 in BF (n = 63), breed was significant (*p* < 0.001), meaning Herefords were more likely to have a case. DLWG between animals (n = 110) that had a case (0.521 kg/d, SD 0.162) and not (0.522 kg/d, SD 0.121) was not significant (*p* = 0.606), treatment group was not significant (*p* = 0.639) and gender was not significant (*p* = 0.309).

### 3.4. Costs of Both Treatments 

Direct costs for one grazing season were analysed for the products used in this trial; these are dependent on the farm handling system, labour and stockist of drugs (Table 4). 

## 4. Discussion

The primary outcome of this trial was cumulative incidence of cases in each treatment group of two separate Cypermethrin products, pour-on or ear tags. There was no significant difference with only four cases difference between the treatment groups. 

The environment in which these animals were kept had a very high level of challenge. Despite being in separate groups, they were all within a mile radius of the farm in paddocks, surrounded by woodland and hedgerows, the ideal fly breeding grounds [18]. Despite both products being used at the higher licenced doses, (pour-on every 6 weeks and two ear tags per animal) there were still 54 cases (27.7% of animals in the trial). Potentially, the environmental challenge was too high as reported in Tarry (1985) [14] and may have overridden any effect the products may have had.

The effect of the Cypermethrin on fly populations has been previously studied [12,13,14], however in this trial there was no negative control group. This was due to the ethical considerations of having to leave a group of animals deliberately unprotected, especially as the farm had a history of high levels of disease. Due to this reason, we were unable to evaluate Cypermethrin’s effect on its own.

Application of these products involves a one-off ear tag application or several repeat treatments with a pour-on preparation. It is crucial it is done at the start of the season as the product works by killing flies on contact with the hair and skin [12]. They work on the principle of reducing the fly population, which involves the need for flies to land on the animal to come into contact with the product. Once fly populations have built up it is very hard to reduce them with late administration of the product. It is unknown whether mixing the two treatment groups had any cross over effect due to animals in the TAG group getting exposure to the pour-on product, from direct contact or licking of the PON animals. 

Two breeds were included on analysis, BF and HEX. HEX were significantly more likely to have a case and this supports the literature that white-faced breeds, are more prone to contracting IBK. Possibly due to the pigment having a protective effect, however evidence is limited on this theory [2,18]. There has been recent work done on flies landing on horses with uniform coloured sheets or striped sheets, more flies landed on horses with uniform colour than striped [19]; this could be transferred to the black and white markings of a Friesian’s face versus the solid white faces of the Hereford’s. 

Age at turnout was also significant; 78% of the cases recorded were in animals under 12 months old. This supports historic literature that younger animals are at a higher risk of cases, thought to be due to lack of immunity [1,2]. However, Pugh (1986) [20] reported a drop in immunity after nine months from exposure. In this trial, there were still 12 cases in animals over 12 months, into their second grazing season, potentially due to a drop in immunity or overwhelming environmental challenge. These results could help identify animals at an increased risk of IBK and be used to target treatment of pesticides. This selective treatment may help reduce pesticide usage and future work needs to be done to determine if it would be effective.

IBK is a painful condition and will affect DLWG in growing animals [7,8,21]; the DLWG was calculated from turnout to point of sale or housing. The DLWG of animals with cases during the trial was only 6 g/d lower on average (0.541 kg/d) and not significantly different to animals without (0.547 kg/d). The lack of a DLWG difference could reflect effective treatment of the cases and also the length of time between the two weight measurements. IBK will stunt growth whilst there is pain during active infection, causing a temporary lack of weight gain. This is usually short-lived if treatment is quickly administered. This is in contrary to Funk (2014) [8] who found a seven kilo difference in yearling weights between animals which had a case at weaning. To see the true impact of IBK on DLWG, animals would have needed to be weighed much more frequently throughout the grazing season, to obtain DLWG measurements for specific time periods of infection. This would help identify periods of temporary lack of weight gain rather than no difference in DLWG over the whole grazing season.

The DLWG measurement could have potentially been affected by other factors including endoparasites; however, these were controlled for by long acting Cydectin being administered at turnout, then for animals kept after September another anthelmintic was administered. As nutrition, all animals were rotated regularly across paddocks; the younger animals were fed concentrates for part of the summer, potentially impacting DLWG figures. As well as causing a potential transmission route from close contact with ocular secretions between infected animals, although this has been reported to be a minimal transmission route [22]. Pre-weaning disease was not recorded in this study, so it is unknown if animals with lower DLWG may also have been impacted by historic disease. 

When DLWG was looked at between treatment groups (n = 190), there was no significant difference in DLWG between the groups. It is thought that handling is a stressful event [23] potentially able to impact DLWG and carcass quality [24]. With pour-on preparations needing to be given every six weeks, this will mean extra handling for these animals. However, in this study the pour-on treatments were often coupled with another reason for handling animals so the effect, if any had been found, would not have been avoidable.

Overall, there was no significant difference between products in the case incidence of IBK. Costs of the products vary (see Table 4) and both products are licenced for fly control. Farmers should use whichever is suitable for their management systems, i.e., farmers with beef animals grazing extensively, with minimal handling facilities may prefer tags as it is a one-time application. For animals that are handled more frequently for other management reasons, pour-on can be applied during these handling events. Pour-on was more expensive but largely dependent on time and labour; efficient handling systems would reduce this. Product selection should be dependent on compliance with management systems and very importantly is applied at turnout before fly populations are established. The effect of IBK on DLWG is recognised but not seen with the data from this study; continual weight monitoring could help pin point the effect of IBK more clearly.

## 5. Conclusions

Overall, there is no significant difference between Cypermethrin pour-on or tag preparations in the prevention of IBK. The action of Cypermethrin alone is still unknown due to no negative control in this study. IBK did not significantly affect DLWG in animals that had cases and those that did not, in this study. Younger animals are significantly more likely to have a case of IBK and white-faced breeds, specifically the Hereford in this study, are significantly more likely to have a case of IBK. 

## Figures and Tables

**Table 1:** Overall numbers of animals and infectious bovine keratoconjunctivitis (IBK) cases in six different management groups.

Group	1	2	3	4	5	6	Total
Number of animals	49	36	32	23	27	28	195
PON *	26	18	16	11	13	14	97
TAG *	25	18	16	12	14	14	98
Average (SD) weight at turnout (kg)	126.4 (SD 20.0)	183.1 (17.9)	243.5 (32.9)	316.3 (55.9)	329.6 (43.1)	303.2 (45.9)	231.3 (85.5)
Average (SD) age at turnout (months)	5.3 (1.5)	7.3 (2.1)	11.4 (2.1)	13.9 (2.5)	15.4 (2.3)	17.4 (2.4)	10.8 (4.9)
Breed – BF **	37	14	13	0	19	28	111
Breed – HEX **	12	22	19	23	8	0	84
Number of IBK cases PON	8	8	5	2	1	6	30
Number of IBK cases TAG	11	7	2	1	0	3	24

* Ear tag (TAG) and Pour-on (PON). ** British Friesian (BF), Hereford crosses (HEX).

**Table 2:** Number of infectious bovine keratoconjunctivitis (IBK) cases by breed and age.

Group	IBK Cases		Total
	<12 Months	>12 Months	
Breed BF *	13 (n = 63)	10 (n = 48)	23
Breed HEX *	29 (n = 50)	2 (n = 34)	31
Total	42	12	

* British Friesian (BF), Hereford crosses (HEX).

**Table 3:** Daily live weight gain (DLWG) differences between each factor and significance values.

Factor		DLWG (kg/d) Mean		Significance
		Mean	SD	
**Treatment Group**	TAG * (n = 95)	0.559	0.167	*p* = 0.215
	PON * (n = 95)	0.531	0.137	
**Case (IBK)**	Y (n = 53) ^1^	0.541	0.163	*p* = 0.739
	N (n = 137)	0.547	0.150	
**Breed**	HEX ** (n = 84)	0.581	0.181	*p* = 0.005
	BF ** (n = 106)	0.517	0.120	
**Sex**	F (n = 99)	0.508	0.114	*p* = 0.001
	M (n = 91)	0.586	0.179	
**Age under 12 Months**	Under 12 months (n = 110)	0.522	0.137	*p* = 0.007
	Over 12 months (n = 80)	0.577	0.168	
**End Month**	September (n = 95)	0.574	0.180	*p* = 0.236
	November (n = 95 Excl SR ^2^)	0.516	0.114	

**^1^** one less case, as one of the dead calves had a case earlier in the trial. * Ear tag (TAG) and Pour-on (PON). ** British Friesian (BF), Hereford crosses (HEX). **^2^** Swedish Red (SR).

**Table 4:** Direct costs involved for both treatment groups in the study.

Treatment	PON *	TAG *
Dose per animal	£ 0.98 × 98 ^1^	£ 7.06 × 99 ^1^
Number of treatments	4	1
Labour cost per extra treatment after turnout	£ 300 ^2^	£ 0
Labour cost to remove ear tags	£ 0	£ 16.80 ^3^
Total	£ 996.04	£ 715.74
Cost per animal	£ 10.16	£ 7.23

**^1^** Prices per animal subject to change from different stockists, price for products used in this trial, excluding VAT. **^2^** Labour costs worked out at 10 hours needed to treat all animals in PON group and paid three workers at £ 10 p/h. **^3^** Labour cost to remove ear tags worked out on a minute per animal (one person) to remove ear tag, at housing or before sale. * Ear tag (TAG) and Pour-on (PON).
